# The Value of the COVID-19 Yorkshire Rehabilitation Scale in the Assessment of Post-COVID among Residents of Long-Term Care Facilities

**DOI:** 10.3390/healthcare12030333

**Published:** 2024-01-28

**Authors:** Łukasz Goździewicz, Sławomir Tobis, Michał Chojnicki, Katarzyna Wieczorowska-Tobis, Agnieszka Neumann-Podczaska

**Affiliations:** 1 Geriatric Unit, Department of Palliative Medicine, Poznań University of Medical Sciences, 61-245 Poznań, Poland; 2 Department of Occupational Therapy, Poznań University of Medical Sciences, 60-781 Poznań, Poland; 3 Department of Immunobiology, Poznań University of Medical Sciences, 60-806 Poznań, Poland; 4 Department of Infectious Diseases, Józef Struś Hospital, 61-285 Poznań, Poland; 5 Department of Human Nutrition and Dietetics, Poznań University of Life Sciences, 60-624 Poznań, Poland

**Keywords:** COVID-19 Yorkshire Rehabilitation Scale, long-term care, post-COVID-19

## Abstract

The COVID-19 Yorkshire Rehabilitation Scale (C19-YRS) is a patient-reported outcome measure designed to assess the long-term effects of COVID-19. The scale was validated and is commonly used in the general population. In this study, we assess the utility of the C19-YRS in evaluating the post-COVID burden among residents of long-term care facilities with a mean age of 79. C19-YRS and Barthel index evaluations were performed among 144 residents of long-term care facilities reporting new or worsened symptoms or functioning three months after convalescence from COVID-19. The C19-YRS-based screening showed that 70.9% of COVID-19 convalescents had ≥1 complaint three months after recovery. The highest C19-YRS-scored symptoms (indicating a higher burden) were breathlessness, fatigue, and cognitive and continence problems; however, symptomatology was very heterogeneous, revealing a high complexity of the disease in older persons. The mean total C19-YRS score was higher in hospitalized patients (n = 78) than in the outpatient group (n = 66) (*p* = 0.02). The functioning subscale of the C19-YRS strongly correlated with the Barthel index, with r = −0.8001 (*p* < 0.0001). A moderately strong correlation existed between retrospectively reported C19-YRS-based functioning and the Barthel index score reported before illness (r = 0.7783, *p* < 0.0001). The C19-YRS is instrumental in evaluating the consequences of COVID-19 among long-term-care residents. The assessment allows for a broad understanding of rehabilitation needs.

## 1. Introduction

Older people, or those with immunodeficiencies and/or chronic diseases, remain at high risk of severe COVID-19, even in a time of the dominance of SARS-CoV-2 Omicron sublineages with less virulence compared to those present at the beginning of the pandemic [1]. Institutional long-term care, i.e., nursing homes and social assistance facilities, are places in which all these factors cumulate, making the community of residents, older people living with advanced physical and/or cognitive disabilities, one of the populations most vulnerable to COVID-19. Long-term care facilities became hotspots for COVID-19 spread and mortality due to increased transmission rates [2,3,4]. While SARS-CoV-2 Omicron sublineages are unlikely to cause an excess of severe COVID-19 similar to that of the SARS-CoV-2 Delta variant, hospital admissions and deaths are still frequent, i.e., reported in about 4–5% of residents [5]. Subsequent outbreaks of COVID-19 in long-term care facilities may sustain strain on residents, personnel, and the healthcare system [6,7]. However, while populational vaccinations decreased the risk of the severe clinical course of COVID-19, they were insufficient to prevent infection outbreaks in long-term care facilities [7,8].

The COVID-19 burden does not end with convalescence. Health deficits may persist for months after the illness and substantially impact patients’ functioning. Post-COVID, also called long-COVID or post-acute sequelae of SARS-CoV-2 infection, was included in the ICD-10 classification in September 2020 and is defined by the World Health Organization as a condition that occurs in individuals with a history of probable or confirmed SARS-CoV-2 infection, usually three months from the onset of COVID-19, with symptoms that last for at least two months. Symptoms, which most commonly include but are not limited to fatigue, shortness of breath, and cognitive dysfunction, cannot be explained by an alternative diagnosis [9]. The literature lists over 200 different symptoms [9,10]; however, no minimal number of symptoms is required for diagnosis at present [9]. Attempts to develop a new post-COVID definition revealed that the algorithm needs to incorporate the symptoms and characteristics of patients, e.g., sex, age, and comorbidities [11]. Patients living in long-term facilities are an example of a population in need of determining the burden of post-COVID and guiding care and rehabilitation programs.

It is well established that the functioning of COVID-19 convalescents living in long-term care facilities, assessed by the standard activities of daily living (ADL) questionnaire, is impaired months after the illness [12,13]. Assessment tools like the Barthel index, which is frequently used as an administrative evaluation of patients in nursing and social assistance homes, capture functioning without quantifying the whole dimension of post-COVID syndrome. This may limit the perception of the healthcare needs of patients. A comparison of the COVID-19 Yorkshire Rehabilitation Scale (C19-YRS) with the EuroQoL 5D-5L, a health-related quality of life assessment tool, showed higher symptom detection with a disease-specific scale and agreement in shared items [14]. Proper tools that allow for the screening of post-COVID syndrome are needed for tailored interventions in patients with post-COVID. Patients of an advanced age and with multimorbidity frequently report post-COVID symptoms, i.e., fatigue, pain, depression, and cognitive dysfunction. It is challenging to distinguish between post-COVID and other clinical entities. Minimally, the evaluation must include at least a comparison of possible syndrome manifestations with health and functioning before the illness. Retrospective assessment of pre-COVID-19 symptoms or functioning reported by patients can be biased.

The pandemic had a substantial impact on residents of long-term care institutions which is not only measured by the number of infections and deaths but also adverse effects on physical and emotional functioning [15,16,17]. Knowledge about post-COVID in older adults, especially those with the most advanced age, complex disabilities, and frailty, is scant [18]. This also concerns the population of residents of long-term facilities who experience rapid and significant functional decline following infection and disabilities persisting from a few to several months [12,13,19]. Investigating delayed COVID-19 effects on long-term care residents requires careful attention to their intricate health issues and varied physical, psychological, and social needs.

As mentioned before, the C19-YRS is a patient-reported outcome measure assessing the long-term effects of COVID-19. The scale encompasses 22 items divided into symptom severity, functional disability, and global health score and provides a comprehensive insight into the persisting symptoms of post-COVID. The clinical utility and psychometric attributes of the C19-YRS were appraised in an observational study of patients with post-COVID attending rehabilitation clinics (n = 187). The analysis employed classical psychometric methods, encompassing data quality, scaling assumptions, targeting, reliability, and validity assessments. The results indicated that the internal consistency was high, with good concordance between the overall perception of health and the patients’ reports of symptoms, functioning, and disability [20]. After the study, the C19-YRS was largely adopted in clinical practice, and a modified version (C19-YRSm) was devised as a patient-reported outcome measure for post-COVID syndrome which has been validated for patient assessment and monitoring in this context [21,22]. In summary, the C19-YRS was studied in populations of individuals recovering from COVID-19, particularly those experiencing lingering symptoms. Through this scale, healthcare professionals can garner a more profound understanding of SARS-CoV-2’s enduring impacts, aiding in enhancing rehabilitation approaches [23,24,25,26]. The C19-YRS had not yet been studied in a population of patients of the most advanced age. Frailty and functional dependency in older patients make them more susceptible to severe and critical conditions, often exhibiting complex and atypical symptoms in sequelae [27,28]. This necessitates a comprehensive approach to their assessment and care management. The C19-YRS captures a broad range of symptoms, functioning items, and overall health, has validated reliability, and focuses on patient-reported outcomes [20,21,22,29].

In this study, we assessed the utility of the C19-YRS to evaluate the post-COVID burden among residents of long-term care facilities. We correlated the total C19-YRS score with the Barthel index to assess the agreement between two measures: the COVID-19-specific and standard measures used in long-term care facilities. In addition, we assessed agreement between retrospectively assessed functioning and the Barthel index assessed before the illness, which is routinely and regularly evaluated in every patient in long-term care facilities in Poland.

## 2. Materials and Methods

### 2.1. Study Design

This was a cross-sectional study utilizing some historically reported data. The study was conducted in four social assistance facilities and one nursing home in Wielkopolska, Poland. SARS-CoV-2 infection was confirmed using PCR testing from nasopharyngeal swabs. All enrolled residents of long-term care facilities were COVID-19 convalescents who reported worsening (including the presence of new symptoms) health or/and functioning three months after their recovery from COVID-19. The screening for the worsening was performed using the C19-YSR. Collected characteristics included sex, age at the time of illness, and COVID-19 course, which was characterized as requiring hospitalization or not. A Barthel index assessment was conducted parallel to the C19-YRS assessment. All assessments were performed three months after recovery from COVID-19.

The C19-YRS questionnaire was obtained from Rory J. O’Connor, Leeds General Infirmary, Leeds, United Kingdom, and translated into Polish. The C19-YRS has four subscales: symptom severity (including the most common symptoms of post-COVID), functional disability, additional symptoms, and overall health. The severity of every symptom (breathlessness, cough, swallowing/nutrition, fatigue, continence, pain/discomfort, cognition, anxiety, depression, and post-traumatic stress disorder (PTSD)), five functional disabilities (communication, mobility, personal care, other ADL, and social role), additional symptoms (palpitations, dizziness/falls, weakness, sleep problems, fever, and skin rash), and overall health were evaluated at scale from 0 to 10 each, where 0 indicated no symptom/disability and 10 indicated extreme symptom severity/disability. Routinely, the C19-YRS includes item evaluation at assessment and before COVID-19, i.e., patients rated all symptoms, functioning, and overall health pre-COVID-19 and at the assessment. All patients reporting impairment in ≥1 item or an additional symptom were included in the analysis.

Patients or their legal representatives gave informed consent before data collection. This study was authorized by the Ethics Committee at the Poznan University of Medical Sciences (approval note KB-247/21).

### 2.2. Data Analysis and Interpretation

The total C19-YRS scores were calculated as the sum of all subscores (22 items). Data were analyzed descriptively, with continuous variables presented as means with SD values and as counts and percentages in the case of nominal variables. Symptom severity and functional impairment assessed by the C19-YRS were considered severe when the mean subscale sore was ≥6, moderate in the range from 3 to 5.9, and mild when <3 [29]. Continuous variables in subgroups of patients were compared using a one-way ANOVA. Pearson’s correlation was used to analyze the association between the Barthel index and the total C19-YRS score or its functioning subscale. To assess agreement between C19-YRS-based functioning retrospectively reported by patients and functioning reported before COVID-19, we assessed the correlating retrospective assessment of functioning performed three months after the illness with the Barthel index score values assessed before COVID-19. Correlations were interpreted based on the method of Chan, Y.H., 2003 [30]. Values *p* < 0.05 were considered statistically significant. Statistical analysis was performed using MedCalc 22.013 statistical software (MedCalc Software, LLC, Ostend, Belgium). Data were visualized using MedCalc software or Microsoft Office Package.

## 3. Results

### 3.1. Patients

In total, 203 patients were screened for the presence of symptoms of worsening functioning or the presence of new symptom(s) three months after COVID-19. Among them, 144 patients (70.9%) declared during the C19-YRS-based screening at least one new complaint or worsening symptoms, functioning, or overall health compared to the period before COVID-19. Participant characteristics and two main analyzed measures are shown in Table 1. Patients aged ≥80 years comprised 52.1% and patients aged ≥65 years comprised 91.6% of the overall population. None of the patients were vaccinated against COVID-19.

### 3.2. Post-COVID Burden Evaluated Using the COVID-19 Yorkshire Rehabilitation Scale

The mean (±SD) total C19-YRS score was 51.2 (±31.2) (Table 1). Women had a higher mean total C19-YRS score than men (57.1 ± 30.4 vs. 42.4 ± 30.4, *p* = 0.005). The total C19-YRS score was higher in hospitalized patients than those who remained in a long-term care facility during the illness (56.9 ± 31.4 vs. 44.7 ± 29.8, *p* = 0.0190); however, there was no difference between the mean Barthel index scores in groups of hospitalized and non-hospitalized patients (53.6 ± 33.5 vs. 61.3 ± 33.2, *p* = 0.1750).

Based on the mean C19-YRS symptom subscale, 3.5% of patients (n = 5) had severe symptoms, 16.7% had moderate symptoms (n = 24), and 79.9% had mild symptoms (n = 115). Severe functional impairment was present in 30.6% of patients (n = 44), moderate functional impairment was present in 31.9% (n = 46), and mild functional impairment was present in 37.5% (n = 54). Table 2 shows symptoms, functioning, overall health, and additional symptom subscores. The mean symptom and functional subscores were higher in the hospitalized patient group than in non-hospitalized patients (Table 2). These two groups had no difference in overall health and additional symptom subscales (*p* = 0.9).

**Figure 1:**
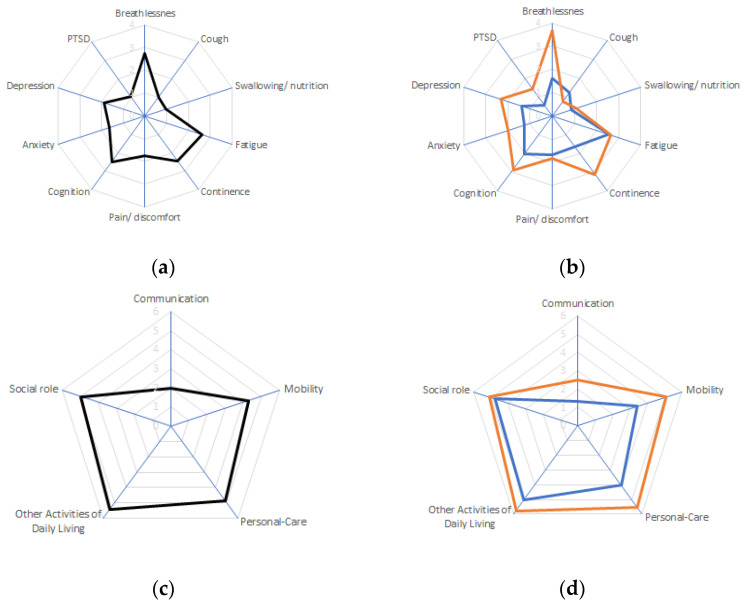
The mean COVID-19 Yorkshire Rehabilitation Scale item scores. Symptom scores overall (**a**) and in subgroups of patients (**b**) hospitalized due to COVID-19 (red) or with a course of the disease not requiring hospitalization (blue). Functioning scores overall (**c**) and in subgroups of patients (**d**) hospitalized due to COVID-19 (red) or with a course of the disease not requiring hospitalization (blue). Higher scores indicate higher symptom severity or highgreater impairment of functioning. The range of scores was from 0 to 10. PTSD, post-traumatic stress disorder.

The symptoms with the highest severity in the overall population were breathlessness, fatigue, cognition disturbances, and continence (Table 2 and Figure 1a). The most affected functioning areas were other ADLs, social roles, and personal care (Table 2 and Figure 1c). Among other symptoms, the highest severity was reported for weakness, sleep problems, and dizziness/falls (Table 2). Symptoms that were more severe in the hospitalized than in the non-hospitalized group were breathlessness, continence, depression, and PTSD (Table 2 and Figure 1b). Patients who were hospitalized due to COVID-19 had a higher impairment of mobility, personal care, and communication than patients who spent the illness in a social assistance facility or nursing home (Table 2 and Figure 1d). There was no difference in overall health perception between hospitalized and not-hospitalized groups, and the only additional symptom that was more severe in the non-hospitalized group than in the hospitalized group was skin rash (Table 2).

### 3.3. Correlation between Assessment Tools

**Figure 2:**
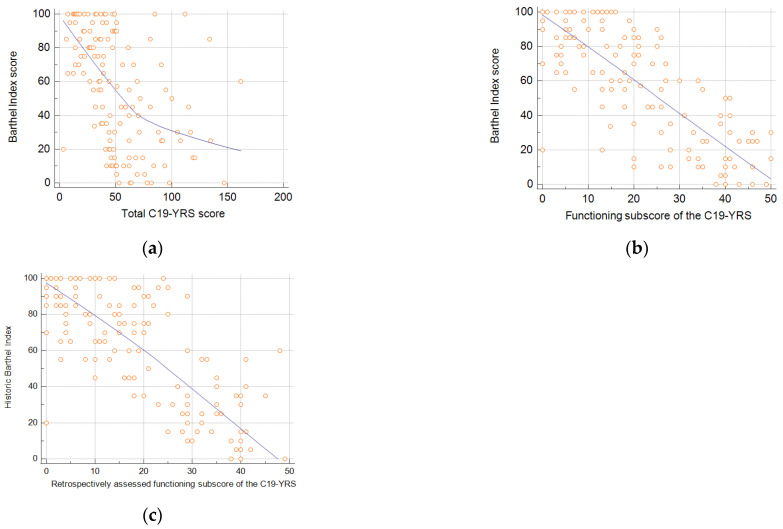
Correlation between total C19-YRS score and the Barthel index (**a**), functioning subscale of the C19-YRS and the Barthel Index (**b**), and retrospective assessment of functioning subscale of the C19-YRS and historic Barthel index assessed before SARS-CoV-2 infection (**c**).

The total C19-YRS score was fairly correlated with the Barthel index, with r = −0.4749 (95% CI: −0.5925 to −0.3376) (*p* < 0.0001) (Figure 2a). There was a very strong correlation between the sum of the functioning scores of the C19-YRS and the Barthel index with r = −0.8001 (95% CI: −0.8521 to −0.7324) (*p* < 0.0001) (Figure 2b). Finally, to assess agreement between patients reporting functioning status which, with the C19-YRS, is reported at assessment and retrospectively for before COVID-19, we correlated these answers with the Barthel index scores recorded before infection as part of routine assessment. There was a moderately strong correlation between the retrospectively reported functioning subscale of the C19-YRS and the historic Barthel index, with r = −0.7783 (95% CI: −0.8359 to −0.7038) (*p* < 0.0001).

## 4. Discussion

We are in a time of intensified research on post-COVID syndrome; however, data available for older adults are scant [31,32,33]. Here, we screened for post-COVID symptoms and impaired ADLs in the group of COVID-19 convalescents living in long-term care facilities. About half of the patients reporting new symptoms or worsening of functioning were over 80 years old. These patients are rarely included in studies assessing the post-COVID burden [18].

Functioning is one of the primarily impaired aspects of post-COVID sequelae and was extensively studied before in a population of residents of long-term facilities [12,13,19]. Older patients more frequently reported mobility and ADL impairments [23]. The current results confirm that the highest scores, indicative of impairment, were attributed to different functioning items compared to symptom items. Almost every third patient had severe functional impairment, highlighting the need for rehabilitation. Similar to the earlier observations of Zhang et al., 2023 [24], the female sex was associated with the presence of post-COVID symptoms.

A fair correlation existed between the total C19-YRS score and the Barthel index, but a robust agreement existed between the functioning subscore and ADL measure. This indicates that the C19-YRS itself captures a broad view of post-COVID-associated dysfunction. This may result in more positive screening results, as shown previously [14]. The use of the C19-YRS for screening post-COVID symptoms in the group of patients ≥ 55 years old resulted in 81.2% of patients reporting ≥ 1 symptom. The COVID-19-specific measure allowed for broader symptom detection than other measures. Interestingly, in our study, the standard ADL measure used in long-term care facilities did not capture the difference in functioning between hospitalized and non-hospitalized patients, while the C19-YRS did. Both symptoms and functional impairment were more pronounced in patients who required a hospital stay due to COVID-19. Patients who needed hospital admission demanded more health resources after the acute infection [26].

In our study, severe symptoms were reported by only a few patients, unlike the previous study [29]. For the first time, we used the C19-YRS to screen a population of advanced-age persons, whereas earlier, general practitioners referred adult patients (men age 47) to a multidisciplinary rehabilitation service [29]. These differences largely contribute to our cohort’s lower severity of symptoms compared to earlier experiences with the C19-YRS. The sampling method largely affects the subscales values recorded, e.g., the random sampling of patients (mean age 67) without severe cognitive and functional impairments, based only on the history of COVID-19 but not the presence of worsening symptoms, functioning, or overall health resulted in lower scores than those reported here [34]. The questionnaire allows one to capture a heterogeneous spectrum of symptoms. In line with earlier reports about the post-COVID manifestation in older adults, fatigue and dyspnea were the most prevalent [24,33]. In our study, breathlessness was the most severe among patients hospitalized due to COVID-19, whereas fatigue severity was similar to that of non-hospitalized patients. Respiratory failure during COVID-19 requiring hospitalization correlated with respiratory symptoms in the year after acute SARS-CoV-2 infection [35]. Breathlessness-associated hypoxia, in combination with long-term care immobilization and social isolation, may result in cardiovascular and neurological complications [36]. Timely respiratory rehabilitation intervention may improve the prognosis. Respiratory rehabilitation benefits pulmonary function, quality of life, and anxiety in older persons with COVID-19 [37]. Another important finding is a relatively high incontinence severity in the studied group. Awareness about the association between SARS-CoV-2 infection and this symptom is low, leading to inadequate early management. The underlying mechanism is poorly understood and possibly involves a low-grade bladder infection, autonomic nervous system dysfunction, pudendal nerve neuropathy, and weakened musculoskeletal systems impacting pelvic floor function [38,39]. It should be stressed that continence (and also swallowing, fever, and skin rash) were infrequently reported in the C19-YRS validation study, and their contribution to the overall measurement properties of the scale was limited [20]. In our study, incontinence was the fourth most severe symptom, indicating that the assessment may have more value in older age than in middle-aged adults. The only symptom more severe in non-hospitalized patients than hospitalized patients was a skin rash; however, the severity was low. Since post-COVID is thought to be an immune disorder driven by viral persistence, molecular mimicry, and other pathological mechanisms [40], a worsening or new-onset disease, autoimmune condition, or allergic reaction is frequently described in the literature [41,42]. The risk of autoimmune manifestation increased over time since the onset of infection and was independent of the patient’s demographics. The main factor predicting the risk of manifestation was COVID-19 in an outpatient setting [42]. The authors considered that anti-inflammatory treatment during hospital stays may decrease the autoimmune burden of post-COVID. The exact mechanism may protect hospitalized patients from developing a skin rash.

We found a very strong correlation between self-reported functioning and ADL evaluated independently. The Barthel index and ADL score are the most frequently used assessments to evaluate the functional impact of COVID-19 across different contexts [43]. However, there are differences between the functioning subscales of the C19-YRS and the Barthel index, e.g., C19-YRS covers communication and social role, which is missing in the Barthel index [20,44]; there was a very strong correlation between both assessments. The severity of symptoms reported by patients can be biased to some degree by subjectivity in their evaluation and a general perception of the worsening of care associated with practices and policies related to the pandemic. In addition to the correlation of assessments performed simultaneously, the functioning reported by patients in the pre-COVID time strongly correlated with the Barthel index score. This indicates that using the C19-YRS gives reliable results regarding functioning before and after the illness and that recall bias is limited, at least with respect to ADL. The reliability of the retrospective assessment, especially in patients for whom syndrome symptoms may overlap with other comorbidities, was one of the issues requiring investigation [18].

This study had limitations. It was a cross-sectional study that captured the long-term COVID-specific burden three months after recovery from the disease; however, post-COVID is not a stable phenomenon, and symptoms and functioning may fluctuate over time, which was evidenced in the nursing home resident population [16]. The study design limited conclusions about the scale’s sensitivity to change. Due to its cross-sectional nature, this study did not allow for a confirmation of the diagnosis of post-COVID since symptoms should persist for at least two months [9]. It is important to note that the mean scores recorded in different studies largely depend on the sampling method. This study was conducted in the early phase of the pandemic, before the populational availability of vaccines. Receiving a primary vaccination and additional doses was associated with fewer post-COVID symptoms in an older population [24]. This and the evolution of SARS-CoV-2 sublineages need to be considered when interpreting data from cohorts from different times. Advanced-age patients are often frail and have physical limitations, cognitive impairments, and dementia, which could affect their ability to accurately report symptoms using the C19-YRS, as well as exacerbate post-COVID symptoms. This and low digital literacy in the population limits the use of the remote version of the scale [45] since many patients would require assistance in answering questions. The symptoms of comorbidities might overlap with post-COVID symptoms, making it challenging to attribute specific symptoms to the syndrome impact accurately.

## 5. Conclusions

The C19-YRS is instrumental in evaluating the consequences of COVID-19 among long-term care residents. The original version of the measure can be used in populations of patients of an advanced age, or at least incontinence should be assessed due to its high severity in this group. The assessment allows for a broader understanding of post-COVID needs compared to the sole activities of daily living check. Information about symptoms and functioning may guide proper rehabilitation interventions since targeting symptoms has been shown to impact functioning. The C19-YRS pre-COVID-19 assessment of functioning had very good agreement with a baseline ADL assessment, confirming the measure’s reliability in assessing status before the illness.

## Figures and Tables

**Table 1:** Patient characteristics.

Characteristic	Patients (n = 144)
Mean age, years (±SD)	78.7 (±9.4)
Males, n (%)	57 (39.6%)
Clinical course of COVID-19, n (%)	
Not hospitalized	66 (45.8%)
Requiring hospitalization	78 (54.2%)
Mean C19-YRS score (±SD)	51.2 (±31.2)
Mean Barthel index score (±SD)	57.1 (±33.5)

**Table 2:** The COVID-19 Yorkshire Rehabilitation Scale symptom, functioning, overall health, and additional symptom items analyzed in all patients and subgroups of non-hospitalized and hospitalized patients. Higher scores indicate higher symptom severity or greater impairment of functioning.

C19-YRS Subscales/Items, Mean ± SD	All Patients	Non-Hospitalized Patients	Hospitalized Patients	*p*-Value *
Symptom subscale	18.6 ± 17.4	21.7 ± 18.2	14.9 ± 15.9	0.0180
Breathlessness	2.7 ± 3.2	1.6 ± 2.4	3.7 ± 3.5	<0.001
Cough	1.0 ± 2.2	1.2 ± 2.4	0.8 ± 2.0	0.2170
Swallowing/nutrition	1.0 ± 2.5	0.8 ± 2.3	1.1 ± 2.7	0.6270
Fatigue	2.6 ± 3.1	2.6 ± 3.1	2.7 ± 3.1	0.8630
Continence	2.4 ± 3.7	1.6 ± 3.2	3.1 ± 4.0	0.0150
Pain/discomfort	1.8 ± 2.9	1.7 ± 2.7	1.8 ± 3.0	0.7530
Cognition	2.5 ± 3.4	2.0 ± 3.0	2.9 ± 3.7	0.1470
Anxiety	1.7 ± 2.9	1.3 ± 2.6	2.0 ± 3.0	0.1310
Depression	1.9 ± 2.7	1.4 ± 2.5	2.3 ± 2.8	0.0410
PTSD ^1^	1.1 ± 2.4	0.6 ± 1.7	1.4 ± 2.8	0.0320
Functioning subscale	21.5 ± 14.8	24.0 ± 17.2	18.6 ± 14.4	0.0280
Communication	2.0 ± 3.1	1.3 ± 2.6	2.5 ± 3.4	0.0200
Mobility	4.3 ± 3.7	3.4 ± 3.5	5.1 ± 3.7	0.0070
Personal care	4.8 ± 3.7	4.0 ± 3.7	5.5 ± 3.6	0.0150
Other ADL	5.4 ± 3.8	5.1 ± 4.0	5.8 ± 3.6	0.2620
Social role	4.9 ± 4.2	4.8 ± 4.3	5.1 ± 4.3	0.6290
Health overall	5.3 ± 2.5	5.3 ± 2.3	5.3 ± 2.7	0.9630
Additional symptom subscale	5.9 ± 7.6	5.9 ± 7.8	5.8 ± 7.6	0.9440
Palpitations	0.7 ± 1.8	0.8 ± 1.8	0.7 ± 1.8	0.7970
Dizziness/falls	1.3 ± 2.1	1.1 ± 1.9	1.4 ± 2.3	0.3840
Weakness	2.0 ± 2.4	1.8 ± 2.0	2.3 ± 2.7	0.2310
Sleep problems	1.4 ± 2.4	1.5 ± 2.6	1.3 ± 2.3	0.6430
Fever	0.2 ± 0.7	0.2 ± 0.7	0.1 ± 0.7	0.6400
Skin rash	0.3 ± 1.3	0.6 ± 1.8	0.1 ± 0.4	0.0100

* one-way ANOVA for comparison between non-hospitalized and hospitalized groups. ADL, activities of daily living; ^1^ PTSD, post-traumatic stress disorder; SD, standard deviation.

## Data Availability

The data supporting this study’s findings are available from the corresponding author upon reasonable request.
