# Correction: Goździewicz et al. The Value of the COVID-19 Yorkshire Rehabilitation Scale in the Assessment of Post-COVID among Residents of Long-Term Care Facilities. *Healthcare* 2024, *12*, 333

**DOI:** 10.3390/healthcare12151469

**Published:** 2024-07-24

**Authors:** Łukasz Goździewicz, Sławomir Tobis, Michał Chojnicki, Katarzyna Wieczorowska-Tobis, Agnieszka Neumann-Podczaska

**Affiliations:** 1 Geriatric Unit, Department of Palliative Medicine, Poznań University of Medical Sciences, 61-245 Poznań, Poland; 2 Department of Occupational Therapy, Poznań University of Medical Sciences, 60-781 Poznań, Poland; 3 Department of Immunobiology, Poznań University of Medical Sciences, 60-806 Poznań, Poland; 4 Department of Infectious Diseases, Józef Struś Hospital, 61-285 Poznań, Poland; 5 Department of Human Nutrition and Dietetics, Poznań University of Life Sciences, 60-624 Poznań, Poland

## Error in Table

In the original publication [1], there was a mistake in Table 2. The symptom, functioning, and additional symptom subscale values ± SD were placed incorrectly, i.e., in opposite columns. The corrected Table 2 appears below. The authors state that the scientific conclusions are unaffected. This correction was approved by the Academic Editor. The original publication has also been updated.

**Table 2:** The COVID-19 Yorkshire Rehabilitation Scale symptom, functioning, overall health, and additional symptom items analyzed in all patients and subgroups of non-hospitalized and hospitalized patients. Higher scores indicate higher symptom severity or greater impairment of functioning.

C19-YRS Subscales/Items, Mean ± SD	All Patients	Non-Hospitalized Patients	Hospitalized Patients	*p*-Value *
Symptom subscale	18.6 ± 17.4	14.9 ± 15.9	21.7 ± 18.2	0.0180
Breathlessness	2.7 ± 3.2	1.6 ± 2.4	3.7 ± 3.5	<0.001
Cough	1.0 ± 2.2	1.2 ± 2.4	0.8 ± 2.0	0.2170
Swallowing/nutrition	1.0 ± 2.5	0.8 ± 2.3	1.1 ± 2.7	0.6270
Fatigue	2.6 ± 3.1	2.6 ± 3.1	2.7 ± 3.1	0.8630
Continence	2.4 ± 3.7	1.6 ± 3.2	3.1 ± 4.0	0.0150
Pain/discomfort	1.8 ± 2.9	1.7 ± 2.7	1.8 ± 3.0	0.7530
Cognition	2.5 ± 3.4	2.0 ± 3.0	2.9 ± 3.7	0.1470
Anxiety	1.7 ± 2.9	1.3 ± 2.6	2.0 ± 3.0	0.1310
Depression	1.9 ± 2.7	1.4 ± 2.5	2.3 ± 2.8	0.0410
PTSD ^1^	1.1 ± 2.4	0.6 ± 1.7	1.4 ± 2.8	0.0320
Functioning subscale	21.5 ± 14.8	18.6 ± 14.4	24.0 ± 17.2	0.0280
Communication	2.0 ± 3.1	1.3 ± 2.6	2.5 ± 3.4	0.0200
Mobility	4.3 ± 3.7	3.4 ± 3.5	5.1 ± 3.7	0.0070
Personal care	4.8 ± 3.7	4.0 ± 3.7	5.5 ± 3.6	0.0150
Other ADL	5.4 ± 3.8	5.1 ± 4.0	5.8 ± 3.6	0.2620
Social role	4.9 ± 4.2	4.8 ± 4.3	5.1 ± 4.3	0.6290
Health overall	5.3 ± 2.5	5.3 ± 2.3	5.3 ± 2.7	0.9630
Additional symptom subscale	5.9 ± 7.6	5.8 ± 7.6	5.9 ± 7.8	0.9440
Palpitations	0.7 ± 1.8	0.8 ± 1.8	0.7 ± 1.8	0.7970
Dizziness/falls	1.3 ± 2.1	1.1 ± 1.9	1.4 ± 2.3	0.3840
Weakness	2.0 ± 2.4	1.8 ± 2.0	2.3 ± 2.7	0.2310
Sleep problems	1.4 ± 2.4	1.5 ± 2.6	1.3 ± 2.3	0.6430
Fever	0.2 ± 0.7	0.2 ± 0.7	0.1 ± 0.7	0.6400
Skin rash	0.3 ± 1.3	0.6 ± 1.8	0.1 ± 0.4	0.0100

* one-way ANOVA for comparison between non-hospitalized and hospitalized groups. ADL, activities of daily living; ^1^ PTSD, post-traumatic stress disorder; SD, standard deviation.
